# Early life neonicotinoid exposure results in proximal benefits and ultimate carryover effects

**DOI:** 10.1038/s41598-021-93894-2

**Published:** 2021-07-27

**Authors:** Thomas Zgirski, Pierre Legagneux, Olivier Chastel, Lyette Regimbald, Louise Prouteau, Audrey Le Pogam, Hélène Budzinski, Oliver P. Love, François Vézina

**Affiliations:** 1 Université du Québec à Rimouski (UQAR), Rimouski, QC G5L 3A1 Canada; 2 Centre de la Science de la Biodiversité du Québec (QCBS), Montréal, QC Canada; 3 Université Laval, Quebec, QC G1V 0A6 Canada; 4 Centre d’Etudes Biologiques de Chizé (CEBC), UMR 7372 CNRS - La Rochelle Université, France, 79360 Villiers-en-Bois, France; 5 Université de Bordeaux & CNRS UMR-5805 EPOC-OASU, 33615 Pessac, France; 6 University of Windsor, Windsor, ON N9B 3P4 Canada; 7 Centre d’Études Nordiques (CEN), Quebec, QC Canada; 8 Groupe de recherche sur les environnements nordiques (BORÉAS), Rimouski, QC Canada

**Keywords:** Growth disorders, Ecophysiology, Metabolism

## Abstract

Neonicotinoids are insecticides widely used as seed treatments that appear to have multiple negative effects on birds at a diversity of biological scales. Adult birds exposed to a low dose of imidacloprid, one of the most commonly used neonicotinoids, presented reduced fat stores, delayed migration and potentially altered orientation. However, little is known on the effect of imidacloprid on birds growth rate despite studies that have documented disruptive effects of low imidacloprid doses on thyroid gland communication. We performed a $2 \times 2$ factorial design experiment in Zebra finches, in which nestling birds were exposed to a very low dose (0.205 mg kg body $\hbox {mass}^{-1}$) of imidacloprid combined with food restriction during posthatch development. During the early developmental period, imidacloprid exposure resulted in an improvement of body condition index in treated nestlings relative to controls. Imidacloprid also led to compensatory growth in food restricted nestlings. This early life neonicotinoid exposure also carried over to adult age, with exposed birds showing higher lean mass and basal metabolic rate than controls at ages of 90–800 days. This study presents the first evidence that very low-dose neonicotinoid exposure during early life can permanently alter adult phenotype in birds.

## Introduction

Neonicotinoids are among the most used insecticides in the world^1–3^. They act by overstimulating the nervous systems of insects^4^ and are partly implicated in their worldwide global decline^5,6^. Adverse effects of neonicotinoids on higher trophic levels could emanate both directly from the consumption of contaminated food items and indirectly from disruptions of the food supply by affecting the insect populations on which they feed^7^. Because neonicotinoids bind with higher affinity to insects than to vertebrate receptors^4^, to some extent it was purported that neonicotinoids would have limited impacts in vertebrates, but the concentrations applied as seed coatings were known to be high enough to have acutely toxic effects on vertebrates prior to registration. A diversity of non-lethal behavioral and physiological negative impacts have been documented in vertebrates exposed to doses (e.g. 0.02–2000 mg $\hbox {kg}^{-1}$) of neonicotinoids^8–13^. In recent studies, birds exposed to imidacloprid, one of the most commonly used neonicotinoids, showed reduced migratory fat stores, delayed migration and potentially altered migratory orientation^8,9^. Other vertebrate studies have documented that imidacloprid exposure can have disruptive effects on the hypothalamo-pituitary axis and thyroid gland function^11,14,15^ as well as metabolic dysfunction^16^, altered growth^17,18^, reproduction^19^ as well as lipid storage disorders through adipogenesis and lipid accumulation^20–22^.

No studies have examined the effects of neonicotinoid exposure during growth in birds, one of the most energetically demanding stages in vertebrate life cycles^23,24^. Growth limitation can have long-term consequences on adult physiology and fitness-related life-history traits, and thus on adult fitness^25–29^. In agroecosystems, nestlings can be exposed to neonicotinoids directly during agricultural applications or via consumption of coated-seeds, contaminated arthropod prey and drinking water^30–32^. Given the potential disruptive effects of neonicotinoids on thyroid function^11,14,15^, which regulates metabolism and resource allocation during growth^33^, it is surprising that the link between neonicotinoids exposure, metabolism and growth have only been examined in young rodents and avian embryos^17,18,34^. In the latter studies, moderate to high doses of imidacloprid (range: 10 mg $\hbox {kg}^{-1}$–337 mg $\hbox {kg}^{-1}$) resulted in skeletal malformations, neurobehavioral deficits and adverse effects on immunity. However, short and long-term effects of early-life neonicotinoid exposure have not been examined, especially at doses that could be encountered in the environment (but see^35^).

**Figure 1:**
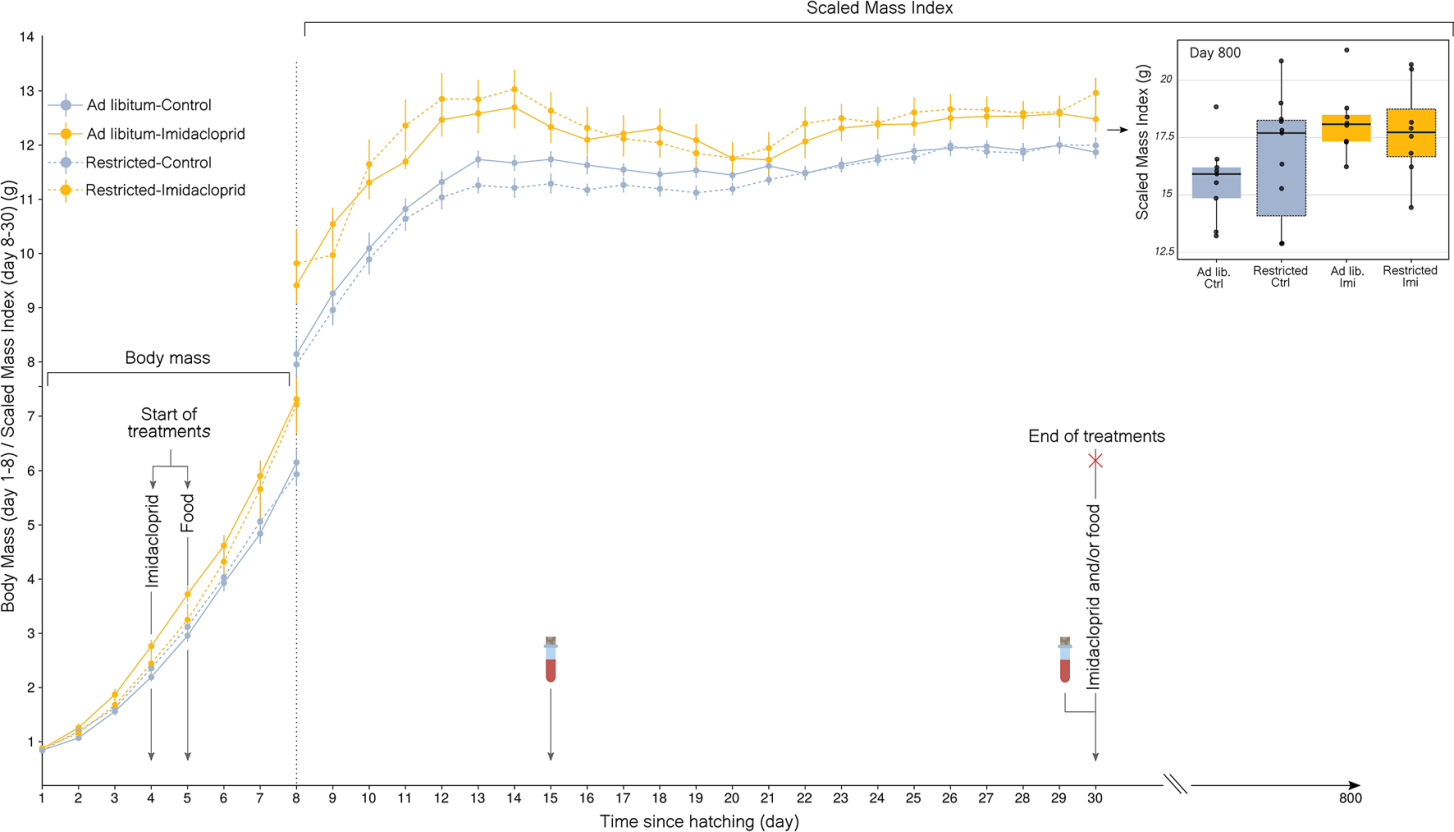
Mean daily body mass (s.e.m.) according to the four treatments (Day 1–30) in the first half of the experiment. Daily administration of imidacloprid (0.205 mg kg body $\hbox {mass}^{-1}$mg/day) occurred from day 4 to 30 after hatching. Food treatment began on day 5. Blood samples were taken on days 15 and 30 (*Ad libitum*-Control n = 18, *Ad libitum*-Imidacloprid n = 13, Restricted-Control n = 19, Restricted-Imidacloprid n = 10). Inserted boxplot shows body mass according to treatments on 800 day old birds. Boxes indicate interquartile range, middle lines indicate median, whiskers show the minimum and maximum values within 1.5 $\times$ the interquartile range while dots outside the whiskers represent outliers (< 1.5$\times$ interquartile range from box).

**Figure 2:**
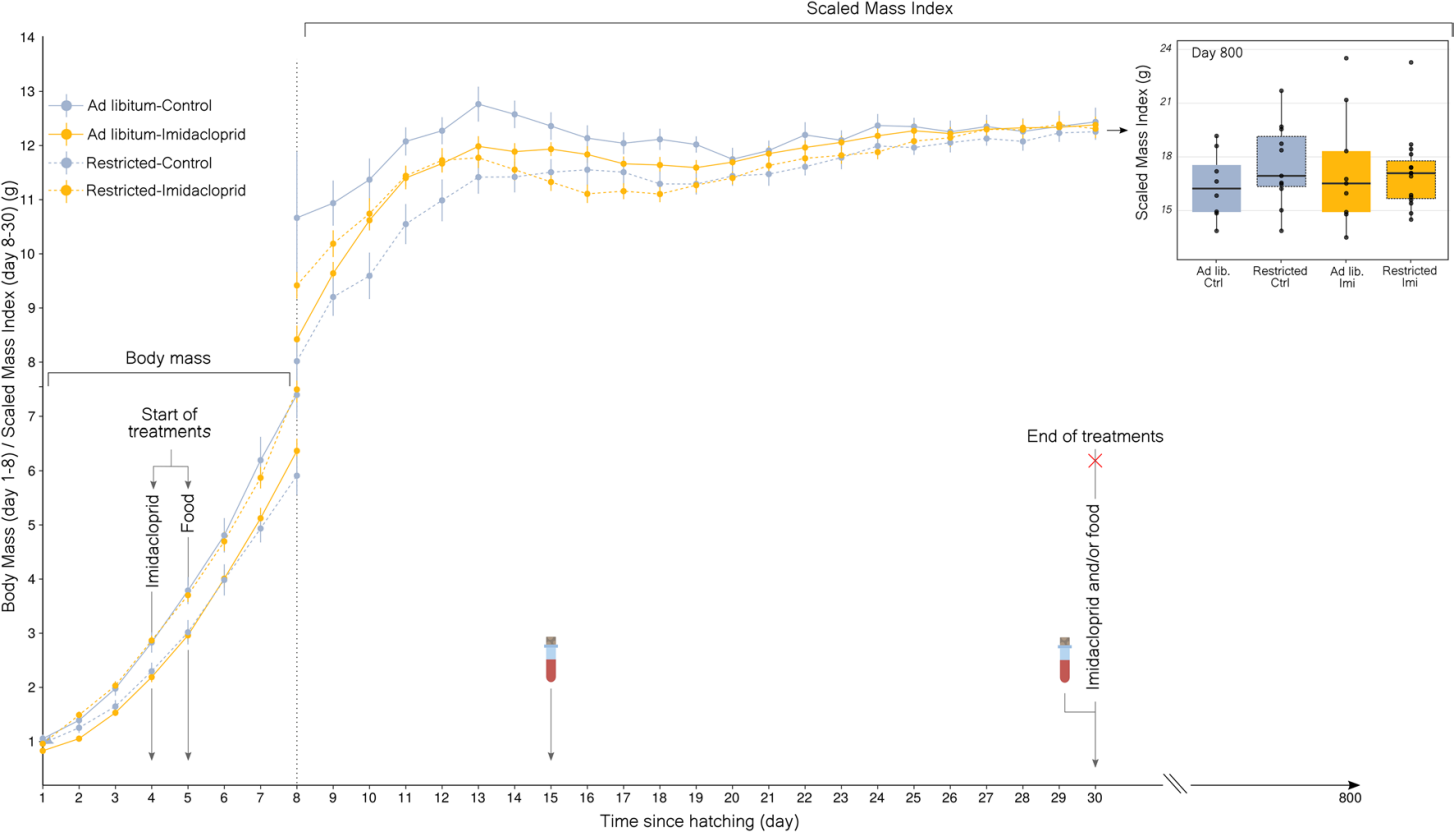
Mean daily body mass (s.e.m.) according to the four treatments (Day 1–30) in the second half of the experiment. Daily administration of imidacloprid (0.205 mg kg body $\hbox {mass}^{-1}$mg/day) occurred from day 4 to 30 after hatching. Food treatment began on day 5. Blood samples were taken on days 15 and 30 (*Ad libitum*-Control n = 18, *Ad libitum*-Imidacloprid n = 13, Restricted-Control n = 19, Restricted-Imidacloprid n = 10). Inserted boxplot shows body mass according to treatments on 800 day old birds. Boxes indicate interquartile range, middle lines indicate median, whiskers show the minimum and maximum values within 1.5 $\times$ the interquartile range while dots outside the whiskers represent outliers ($<1.5\times$ interquartile range from box).

We used a $2\times 2$ factorial experimental design in a model bird species, the Zebra finch (*Taeniopygia guttata*) to examine direct effects of the neonicotinoid imidacloprid on body condition and thyroid function during growth and the long term effects on adult physiology, focusing on body composition and metabolism. We considered two major routes of impact from neonicotinoid application during intensive agriculture: direct pesticide exposure and indirect food reduction (i.e., via a reduction in foraging resources). Zebra finches were exposed daily to a low dose of imidacloprid [0.205 mg kg body $\hbox {mass}^{-1}, 0.5\%$ of the LD50 of House sparrow (*Passer domesticus*) 41 mg $\hbox {kg}^{-1}$] and/or to food restriction during posthatch growth. This dose was comparable to the one used in a passerine, the Red munia (*Amandava amandava*) where the administration of 0.155 mg $\hbox {kg}^{-1}$ of imidacloprid in adults induced thyrotoxicity with potential damaging effects on thyroid cells^11^. The dose used in the current study can be considered to be environmentally relevant for most farmland birds species that incorporate a mix of invertebrates and seeds in their diet during the nestling rearing period ^36^. Birds in our study were exposed to imidacloprid from days 4 to day 30 after hatching (Fig. 1) which represents a total exposure of 0.059 mg of imidacloprid per nestling and is equivalent to two canola seeds treated with imidacloprid (0.03 mg of imidacloprid per seed^9^). The current experiment was conducted on 129 nestlings over 3 months (See “Appendix A” for a visualisation of the birds used over time).

## Results

### Plasma imidacloprid concentration

We first validated that the ingested pesticide was present in nestlings’ blood by measuring imidacloprid concentration in plasma. This was done by pooling samples (to increase plasma volume and detection rate) of individuals from the first and second half of the experiment to obtain two pooled samples (“Appendix A”). Imidacloprid plasma concentrations were 7.92 ng.g $^{-1}$ plasma and 4.19 ng.g $^{-1}$ plasma during the first and second half of the experiment respectively and only trace amounts were detected in controls (one pool: 0.08 ng $\hbox {g}^{-1}$ plasma). To account for this change in imidacloprid concentration over time, data were analyzed with an interaction effect between treatment and period. The number of individuals used in both periods were similar (60 and 69 in the first and second half of the experiment respectively (“Appendix A”). 5 individuals died during the growth experiment (Restricted-Control: 2; *Ad libitum*-Control: 1; Restricted-Imidacloprid:1; *Ad libitum*-Imidacloprid: 1). The body mass, tarsus length and head-bill length of the parents as well as the brood size were also similar in both time periods (All t $\leqslant$ 1.29; All p$\geqslant$ 0.08). However, in the second half-period, *Ad libitum*-Imidacloprid nestlings were lighter than Controls before start of treatments (treatment effect day 1–4 on body mas s$\beta$ = −0.37 ± 0.14, P = 0.023).

**Table 1:** (**a**) Model selection of the effects of treatments (*Ad libitum*-Control, *Ad libitum*-Imidacloprid, Restricted-Control, Restricted-Imidacloprid), Sex, Brood size, Pair order) on scaled body condition residuals (SMI $_{residuals}$) from day 8 to day 30. (**b**) Parameter estimates (slopes [$\beta$] with standard error [*SE*], degrees of freedom [df], t-value and P-value) for top ranked model. Factor level references: Treatment =* Ad libitum*-Control, Period = First half-period. (N observations = 2937; from 129 individuals of 35 pairs used as random factors).

**a**) Model		$N _{p}$	$\bigtriangleup _{AIC _{c}}$	$\omega _{i}$	
SMI $_{residuals}$					
	1. Treatment*Period + Treatment*Day + Brood size	16	0.00	0.43	
	2. Treatment*Period + Treatment*Day + Brood size + Pair order	17	0.40	0.35	
	3. Treatment*Period + Treatment*Day + Brood size + Pair order + Sex	18	1.30	0.22	
	4. Null	5	91.36	0.00	

**b**) SMI $_{residuals}$	$\beta$	$SE$	df	t-value	P-value
(Intercept)	0.43	0.29	42.14	1.50	n.s.
*Ad libitum*-Imidacloprid	0.41	0.28	26.61	1.47	n.s.
Restricted-Control	−0.98	0.26	26.46	−3.81	< 0.001***
Restricted-Imidacloprid	0.88	0.30	25.05	2.91	< 0.01**
Period	0.69	0.27	18.25	2.54	< 0.05*
Day	0.00	0.00	2809	0.42	n.s.
Brood size	−0.15	0.04	38.49	−3.35	< 0.01**
*Ad libitum*-Imidacloprid:Period	−0.87	0.37	19.95	−2.34	< 0.05*
Restricted-Control:Period	−0.49	0.37	18.21	−1.30	n.s.
Restricted-Imidacloprid:Period	−1.36	0.39	19.32	−3.52	< 0.01**
*Ad libitum*-Imidacloprid:Day	0.01	0.00	2809	2.09	< 0.05*
Restricted-Control:Day	0.04	0.00	2809	7.73	< 0.001***
Restricted-Imidacloprid:Day	0.00	0.00	2809	−0.18	n.s.
**Random effects**					
Individual standard deviation	0.54				
Pair standard deviation	0.22				
Residual standard deviation	0.65				

### Direct effects of imidacloprid exposure during growth

Exposure to imidacloprid resulted in higher body condition index (Scaled-Mass Index hereafter SMI^37^) in treated nestlings relative to controls during the first half of the experiment (Treatment effect: $\hbox {F}_{(3;9.99)}$ = 15.07, P $\leqslant$0.001, Fig. 1, Table 1). Since this effect was consistent for both the *Ad libitum* and food restricted groups, imidacloprid exposure resulted in a compensatory effect on growth in restricted birds (Fig. 1). The greatest impact on SMI was found on day 8, where Restricted-Imidacloprid birds were +19.0 $\%$ heavier (SMI) than Restricted-Control birds. Similarly, *Ad libitum*-Imidacloprid birds were +14.6 $\%$ heavier than *Ad libitum*-Control birds. This effect persisted until the end of the experiment at day 30 (close to independence), where Restricted-Imidacloprid and *Ad libitum*-Imidacloprid birds were still +7.5 $\%$ and +4.9 $\%$ heavier than their respective controls. There was a significant interaction between treatment and day but resulted from an effect size (Table 1). This increase in SMI was not associated with a change in structural body size in imidacloprid exposed birds. Total water content was similar for all measured individuals (Mean total water = 10.50 ± 0.29 (n=6) and 10.49 ± 0.13 (n=41) for imidacloprid during the first and second half respectively and 10.37 ± 0.18 (n=13) for the control group in the second-half period) with all statistical results remaining highly non-significant (all F < 0.218; All p-values > 0.65). Food restriction had negative effect on head-bill length in non-exposed birds in the second half-period (food restriction effect on head-bill length $\beta = -0.72 \pm$ 0.17, P $\leqslant$0.001, “Appendix B”). The effect of food restriction was comparable to a previous study that used the exact same procedure^38^. We compared the body mass difference between control and restricted birds from day 5 to day 18 for which similar data was available. Mean difference was 0.90 [±95% CI 0.71–1.09] and 0.70 [± 95% CI 0.57–0.84] for Spencer et al. (2003) and our study respectively. While no statistical differences between the two studies were detected, the impact of food restriction was reduced in our study potentially because additional corn oil was administered to our nestlings. This mitigation effect of imidacloprid exposed birds was more subtle during the second half of the experiment (Treatment effect: $\hbox {F}_{(3;29.76)}$ = 17.27, P $\leqslant$ 0.001) with no effect of imidacloprid treatment and a strong effect of the nutritional stress (Table 1). Plasma triiodothyronine (T3) levels measured at days 15 and 30 and thyroxin (T4) levels measured at day 30 revealed no significant treatment effects on either hormone regardless of the treatment period considered (“Appendix C”).

### Imidacloprid effects in post-fledging and adult birds

We investigated long-term effects of early imidacloprid exposure on body, lean and fat mass using quantitative magnetic resonance on days 90 and 800. We also examined effects on basal metabolic rate measured by respirometry [on day 800 after keeping the birds in optimal nutritional and daylight conditions with no further imidacloprid exposure (see insert in Figs. 1 and 2)].

**Figure 3:**
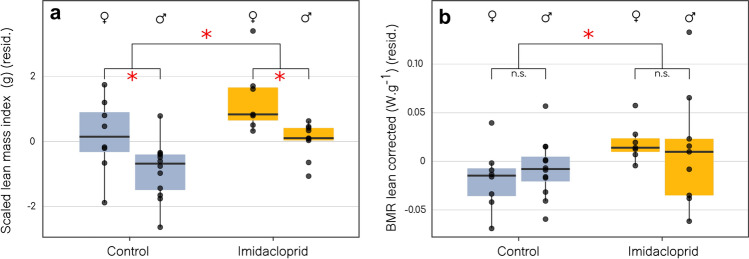
(**a**) Residuals of Scaled Lean Mass Index (day 800) split by sex and early-life treatments (Control in blue and Imidacloprid in orange). Control (n = 20) = *Ad libitum*-Control + Restricted-Control / Imidacloprid (n = 16) = *Ad libitum*-Imidacloprid + Restricted-Imidacloprid). (**b**) Lean mass corrected Basal Metabolic Rate (model residuals) (day 800) according to the sex and the two treatments (Control and Imidacloprid). Specifications of the boxplots are provided in the caption of Figure 1. These results show individuals treated during the first half of the experiment. P-value $\le$ 0.05*.

At day 90 when birds are considered adult and able to breed, we did not detect any residual effect of early imidacloprid exposure and food restriction on SMI (See "Appendix D1 and D2"), which indicates that the birds condition had recovered within 60 days after the end of imidacloprid exposure. The lack of difference in SMI among the groups persisted after two years (or 800 days; the expected maximum lifespan of this species in the wild). However, both at age 90 and 800 days, birds that were exposed to imidacloprid during the first half of the experiment had significantly heavier lean body mass (SLMI, Scaled Lean Mass Index) than controls [Fig. 3A; imidacloprid effect on SLMI $\beta$ = 0.97 ± 0.19, P $\leqslant$0.001 and $\beta$ = 1.11 ± 0.37, P = 0.013 for age 90 and 800 days respectively (see "Appendix E1 and E2"), this effect was not detected in the second half of the experiment]. This increase in SLMI was consistent and independent from the nutritional treatment considered ("Appendix F1 and F2"). Females also had higher lean body mass compared to males irrespective of the period considered ($\beta$ = 0.85 ± 0.21 ; Fig. 3A, no interaction between sex and treatment). Furthermore, Scaled Fat Mass Index (SFMI) was higher in imidacloprid exposed birds at 800 days but only for birds exposed during the first half of the experiment (imidacloprid effect on SFMI: $\beta$ = 1.03 ± 0.41, P = 0.031, "Appendix G1 and G2"). In other words, birds that were exposed to imidacloprid during growth maintained both higher lean and fat mass later in life (see "Appendix H" for summary statistics). This change in body composition was associated with significantly higher lean-corrected basal metabolic rate (BMR) in imidacloprid exposed birds compared to controls (Figure 3B). The effect of early-life imidacloprid exposure on adult BMR was only found in birds exposed during the first half of the experiment (imidacloprid effect on lean mass corrected BMR, $\beta$ = 0.83 ± 0.34, P = 0.030 and $\beta$ = 0.59 ± 0.28 for the second half of the experiment, see "Appendix I1 and I2" for more details).

## Discussion

We have shown that short-term exposure to a very low dose of imidacloprid, one of the most commonly-used neonicotinoid pesticides in the world, can positively impact body condition index during growth and body composition of adult birds. Counterintuitively, imidacloprid enhanced body condition and appeared to fully compensate for the negative effects of growing under a food-restricted environment. Interestingly, those beneficial and compensation effects faded at a lower imidacloprid concentration (i.e. during the second half of the experiment).

Our study is the first to report a positive effect of low imidacloprid exposure on the development and body condition of juvenile birds. This suggests that the influence of very low dose imidacloprid on body condition is prominent only at juvenile stages in birds. Indeed, adult passerine Red munia (*Amandava amandava*) daily exposed to 0.155 mg kg body $\hbox {mass}^{-1}$ of imidacloprid [0.5$\%$ LD50 of Japanese quail (*Coturnix japonica*), 31 mg $\hbox {kg}^{-1}$] over 30 days did not change their body mass^11^. The increase in body condition reported in the current study was not associated with a change in structural body size and is consistent with other studies in rodents where a mass gain was reported in mice and rats exposed to very low doses of imidacloprid (^16^ 0.5 mg  $\hbox {kg}^{-1}$^21^, 0.06 mg.$\hbox {kg}^{-1}$).

During the second half of the experiment (lowest concentration), the short and positive effect of imidacloprid administration on growth rate was considerably reduced with no carry-over effects at the adult stage. Dosing the solution over the course of the experiment and/or individual plasma would have been ideal instead of pooling the samples for the two periods (see methods). However, since the exact same protocol was followed during the first and the second half of the experiment, we are confident that the differences occurred because of the fading of the imidacloprid solution and not from different tested groups. This dose-response could non-exclusively result from either a threshold effect of imidacloprid or from non-linear processes, also known as hormesis, a biphasic toxicological phenomenon characterized by low-dose stimulation and high-dose inhibition^39,40^. At higher doses, the reported effects on physiology or behaviour are apparently always deleterious^8,9,41^. Taken together, the dose-response of imidacloprid exposure on organisms is likely non-linear. Future studies are required to confirm the potential hormetic nature of imidacloprid exposure in vertebrates, as shown in arthropods^39,42^.

Egg yolk thyroid hormones thyroxine (T4) and triiodothyronine (T3) produced already after a few days of incubation^43^ can regulate several key physiological processes such as prenatal development and juvenile growth^33^ and metabolism^44^ for which imidacloprid was found to imbalance their plasma concentrations^11^. However, in our study, plasma T3 and T4 levels were not influenced by imidacloprid exposure nor food restriction. These results are surprising given recent studies reporting higher T4 and lower T3 in Red munias exposed to 0.155 mg kg body $\hbox {mass}^{-1}$ of imidacloprid^11^ and an inverse response (lower T4 and higher T3) to food restriction in domestic fowl^45–48^ and mice^49^. Therefore, the underlying mechanisms still require dedicated studies, since thyroid hormones were apparently not playing a significant role in our system and imidacloprid did not influence the hydric balance of exposed birds. Once adult, exposed birds maintained higher levels of both lean and fat masses. Lean tissues are known to have higher metabolic activity than adipose tissues^50^ and this could partly explain the observed high BMR in adult imidacloprid birds. The influence of imidacloprid on body reserves has been of great interest in human studies as it is thought to have obesogenic effects by promoting lipid accumulation in 3T3-L1 adipocytes^20–22,51^, resulting in increased body fat accumulation. However, such obesogenic cellular mechanisms have not been examined in birds and were beyond the scope of this study.

Our study highlights that a very low dose of imidacloprid can benefit development in birds with long-lasting consequences. Environmental conditions experienced during early life can act directly on adult phenotypes with influences on individual fitness components^26,52^. In this study, the mitigation effect of imidacloprid exposure on SMI was particularly high during the period of rapid linear growth, where energy is known to be mostly allocated toward tissue-synthesis^53^. This ultimately resulted in an accumulation of lean tissues and larger lipid stores in adult birds. Therefore, early-life imidacloprid exposure induced long term effects, which consequently resulted in higher maintenance cost during adulthood^54^. While the development of an optimal phenotype may be constrained by a wide range of environmental conditions, our study revealed that neonicotinoids superimpose a new layer of complexity in resource allocation and that this can be carried over to generate adults living with higher maintenance costs.

## Methods

### Bird husbandry and experimental approach

This study took place at the avian facility of the Université du Québec à Rimouski (UQAR), from November 2016 to March 2019. Birds were kept at a constant temperature of 21 $^{\circ }$C on a 12L:12D cycle during the entire experiment. This experiment was conducted over two consecutive breeding trials (first half: 28-11-2016 to 15-01-2017, second half: 16-01-2017 to 11-03-2017 "Appendix A").

Adults were randomly paired and kept together in 41x42x32 cm in canary breeding cages with an external cardboard nest box attached to the front wire mesh panel. After twelve days, unsuccessful pairs were redistributed. Nest boxes were checked every day between 9:00 and 11:00 during the laying period. Egg cross-fostering was performed to avoid potential maternal effects in egg production, incubation and nestling rearing. Each egg was therefore swapped with an egg from another pair laid on the same date. This procedure was conducted for pairs with the same treatment (intra-group) and between pairs with another treatment (inter-treatments groups). 68,7$\%$ (total=67 pairs) of the breeding pairs succeeded to produce a clutch and 20 pairs produced a second clutch that were included in the experiment (Second clutch effect: $\hbox {F}_{(1;286.2)}$ = 9.10$^{-4}$, P = 0.98). Nine pairs failed between day 1 and 5 after hatching and were discarded from the analysis. Nest boxes were checked twice per day toward the end of the incubation period (12 to 14-days after clutch completion) to determine hatching dates and to associate eggs with nestlings. After hatching, breeding pairs were randomly assigned to one of four treatments, *Ad libitum*-Control, *Ad libitum*-Imidacloprid, Restricted-Control, Restricted-Imidacloprid. Consequently each nestlings in a given nest received the same treatment, resulting in a 2 × 2 factorial design experiment. Water was available *Ad libitum* for all groups. Additionally, electrolytes were mixed to water twice a week. Each nestling was marked with a coloured spot (using food dye to track each individual from a given clutch) from day 1 to day 15 and then fitted with a unique alpha-numeric color band. Birds were weighed every day (0.01 g) before treatment administration. Wing chord, tarsus and head-bill lengths were measured (0.01 mm) every two days starting at age 8. All procedure took place between 09:00 and 11:00. Blood samples were collected in a second round of manipulation, at least an hour after imidacloprid administration between 11:00 and 12:00 am. Blood samples were collected at day 15 and 30 from the brachial vein using a 27-gauge needle and a 70–100 μL heparinized capillary tube which represent <1 $\%$ of the bird’s total body mass. These samples were later centrifuged (10 min at 8,000 rpm; UNICO PowerSpin BX centrifuge C886, UNICO, Dayton, NJ) to obtain plasma samples and hematocrit data. Plasma samples were stored at −20 $^{\circ }$C. After day-30, birds were maintained in captivity (males and females were kept separately in large cages with 2-10 individuals per cage) and fed *Ad libitum* until basal metabolic rate measurements two years later. Research protocols were in compliance with the Canadian Council on Animal Care guidelines and approved by the Université du Québec à Rimouski (UQAR) Animal Care Committee (CPA-67-16-180). The study was carried out in compliance with the ARRIVE guidelines.

### Imidacloprid treatment

Treated birds were exposed daily to imidacloprid (analytical standard, Toronto Research Chemicals (I274990) from day 4 to day 30 using corn oil as a vehicle (organic food grade). The chosen dose, 0.205 mg $\hbox {g}^{-1}$ represented 0.5$\%$ of LD50 of House sparrow (*Passer domesticus*) (41 mg $\hbox {kg}^{-1}$). Control groups only received corn oil. The dosing solution was prepared by directly adding imidacloprid to the oil and then stirring overnight to ensure complete homogeneity. We orally administered 5.13 μL $\hbox {g}^{-1}$ mass of nestlings (for both treated and control groups) using a micropipette with a 100 μL tip cone. Complete swallowing with this technique was confirmed after administration, by observing 20 nestlings (10 controls and 10 imidacloprid). After administration, those 20 nestlings were kept in the dark for 10 minutes. At 5 min and 10 min, the beak gapes were observed to detect any oil regurgitation. From these observations, we confirmed complete swallowing after injection in 100$\%$ cases. The solution was protected from direct light and stored in the dark and kept at 4 $^{\circ }$C during the whole experiment.

To validate and quantify imidacloprid absorption in nestlings, we used on-line Solid-Phase Extraction/Liquid Chromatography coupled to Tandem Mass Spectrometry SPE-LC-MS/MS. As we used the same solution during the whole experiment, we measured imidacloprid concentration in plasma taken during the first and the second half of the experiment (“Appendix A”). We found a reduction in imidacloprid plasma concentration between the first and the second half of the experiment that could have resulted from a degradation in the stock solution during the experiment despite its conservation in the dark at 4 $^{\circ }$C during the entire experiment.

### Imidacloprid chemicals analysis

Remaining blood samples (plasma was first used to measure T3 and T4 concentration in plasma, see below) taken at days 15 and 30 were pooled into three groups: Control birds (*Ad libitum* + food restricted); imidacloprid exposed birds from the first and the second half of the experiment respectively (“Appendix A”). The samples could not be analyzed individually because we did not have enough plasma per pool after the T3 and T4 analyses. We pooled 8 (first half) and 24 (second half) samples per pool in imidacloprid groups (Control n = 27). To dose imidacloprid in the plasma^55,56^, internal standard imidacloprid-d4 was added to 25 μL of plasma. 25 μL intake plasma volume is the lowest acceptable volume to realize the protocol with a good sensitivity (LOQ imidacloprid = 10 pg/g plasma with this method). Plasma samples were pretreated with acetonitrile to precipitate proteins. Before analysis, ultra-pure water was added to the supernatant and extracts were injected to the analytical system composed of a Poroshell 120 SB C18 guard column (5 mm length $\times 2.1$ mm internal diameter, 2.7 μm particle size) (Agilent) used as on-line SPE cartridge. Target compound separation was realized with a Poroshell Phenyl-Hexyl column (100 mm × 2.1 mm, 2.7 μm) (Agilent). Imidacloprid detection was ensured by the 6490 Triple Quad mass spectrometer (Agilent). Finally, we checked the analytical sequence by quality controls and used internal standards to calculate the concentration. Limit of quantification for imidacloprid is 10 pg $\hbox {g}^{-1}$ plasma. Analyses were carried out at the Université de Bordeaux (UMR CNRS 5805 EPOC).

### Food restriction treatment

To experimentally reduce nestling’s growth rate, we manipulated food accessibility to breeding adults. Food restriction occurred from day 5 and stopped at day 30 when Zebra finch nestlings are considered to reach nutritional independence. All groups were fed daily with 50 g of a commercial finch seed mix. However, in the food restricted groups seeds were mixed with buckwheat husks with a volume ratio of 1:3. This method is known to decrease food intake rate in Zebra finch by increasing parental foraging effort, mimicking a poor food resource environment^57^. Ultimately, this can decrease nestling-feeding rates and growth^38^. Breeding pairs were also supplemented with egg food (homogenized hard-boiled chicken eggs with breadcrumbs). *Ad libitum* and food restricted pairs were respectively supplemented with 15g and 6.5g of egg food. Egg food was supplemented for the duration of the breeding process (pairing until chicks were 30 days of age), so that all breeding pairs received 15g until the day of hatching then 6.5g for pairs in the food restricted group.

### Thyroid hormones analyses

Blood samples were taken at day-15 and -30 to test the influence of treatments on nestlings triiodothyronine (T3) and thyroxin (T4) levels. T3 and T4 analyses were performed by radioimmunoassay (RIA) at the Centre d’Études Biologiques de Chizé^58,59^. Total TH levels were assessed in duplicates (20 $\upmu$l plasma) in one run without extraction. Plasma were incubated during 24h with 10000 cpm of the appropriate $^{125}$I-hormones (Perin ELmer, US) and polyclonal rabbit antiserum supplied by Sigma company (US). The bound fraction (hormone linked to antibody) was then separated from the free fraction by addition of a sheep anti-rabbit antibody and centrifugation. After overnight incubation and centrifugation, bound fraction activity was counted on a wizard 2 gamma counter (Perkin Elmer, US). Cross-reactions of T3 antiserum were defined as follows by Sigma: triiodoD-thyroacetic acid 6%, L-thyroxine 0.2%, diiodo-L-thyrosine<0.01%, monoiodo-L-thyrosine<0.01%. Cross-reactions of T4 antiserum were defined as follows by Sigma: triiodothyronine 4%, diiodo-L-thyrosine <0.01%, monoiodo-L-thyrosine <0.01%. Intra-assay variations were respectively 9.20% and 7.19% for T3 and T4. The lowest T3 detectable concentration was 0.42ng.$\hbox {ml}^{-1}$ and it was 0.29 ng.$\hbox {ml}^{-1}$ for T4. Two samples were serially diluted in the assay buffer and their displacement curves were parallel to the standard curve.

### Body composition and metabolic rate

We measured the lean and fat components of body mass non-invasively by quantitative magnetic resonance (EchoMRI, Houston, TX, USA^60^, 10 min procedure for triplicate measurements, see Le Pogam et al. 2020^61^) at 90 days and at around 800 of age as well as basal metabolic rate at around 800 days (mean age = 791 days, min = 758 days, max = 830 days). QMR was only available at the end of period A (21/01/2017).

Adult Basal metabolic rates were measured overnight (average duration: 09:33 ± 25min) simultaneously on four birds. Birds were placed in airtight stainless-steel metabolic chambers (1.5 L) equipped with a perch and a copper constantan thermocouple connected to a Sable Systems TC-2000 thermocouple reader (Sable Systems, Las Vegas, NV, USA) to continuously monitor chamber temperature. Metabolic chambers were placed in a PELT-5 (Sable Systems, Las Vegas, NV, USA) temperature cabinet set at 35 $^\circ$C (within the thermoneutral zone for the species). Birds received dry, CO2-free air at a constant rate of 500 mL.min-1 maintained by mass flow controllers. The air sent to the analyzer alternated automatically between reference air (10min) and chamber air (40min) using a multiplexer (Sables Systems MUX, Sable Systems, Las Vegas, NV, USA). BMR calculations were based on the lowest averaged 10 minutes of VO2 using equation 10.1 from Lighton (2008)^62^. The duration of BMR trials ensured that birds were post-absorptive at the time of BMR measurement (which occurred after 6.28 ± 2.45 h of measurement on average). We converted to energy consumption assuming a respiratory quotient of 0.71 and using a thermal equivalent of 19.8 kJ L $\hbox {O}_{{2}}$ $^{-1}$ and converted units to Watts^63^.

### Statistical analysis

We used the scaled mass index (SMI) as a body condition index (BCI)^37^. The body condition index was obtained from the most correlated structural body measurement to body mass (Scaled Mass Index, SMI), lean mass (Scaled Lean Mass Index, SLMI) or fat mass (Scaled Fat Mass Index, SFMI). The body condition index can be computed as:

$$\begin{aligned} BCI_{i}= M_{i}\left[\frac{L_{0}}{L_{i}}\right]^{b_{SMA}} \end{aligned}$$

where $M_{i}$ and $L_{i}$ are the body mass or the body composition component and the linear body measurement of individual i respectively; $b_{sma}$ is the scaling exponent calculated by dividing the slope from an OLS regression by the Pearson’s correlation coefficient r of M on L; $L_{0}$ is the arithmetic mean value of the linear body measurement. Head-bill was the structural size that most strongly correlated with body mass during the growth period (head-bill mean = 20.55, r = 0.90, P $\leqslant$ 0.001, SMI $b_{sma}$ = 0.88) while tarsus length was the most related structural size metric to body mass at both 90 and 800 days old (Tarsus mean = 16.72, r = 0.27; P $\leqslant$ 0.01, $SMI_{90}$ $b_{sma}$ = 2.68, $SMI_{800}$ $b_{sma}$ = 4.18, $SLMI_{90}$ $b_{sma}$ = 1.51, $SLMI_{800}$ $b_{sma}$ = 2.21, $SFMI_{90}$ $b_{sma}$ = 1.72, $SFMI_{800}$ $b_{sma}$ = 2.82). As Head-bill length was measured every two days during the growth, we interpolated sequential measurements assuming a linear skeletal growth over a 2 days period. We thus obtained an SMI for each day of the experiment from day 8 to day 30.

Before testing the effect of treatments on body condition, we applied different nonlinear functions to our full dataset. Quadratic, logistic and Gompertz functions were tested using AICc. The logistic function best fitted our data ($\bigtriangleup _{AIC _{c}}$ > 2.10) and was retained. We estimated variability in body condition index of individual nestlings from the residuals of the logistic fit relating the scaled mass index and day of all measured nestlings using the ‘nls’ function in R^64^. The adjusted equation was:

$$\begin{aligned} \hbox {SMI }=\frac{11.944}{1 + e^{-(6.367 + 1.676 * day)}} [\hbox {All 3 parameters of the equation were highly significants } (\hbox {all t }\geqslant 20.9, \hbox {all }P < 0.001)] \end{aligned}$$

Residuals from this logistic relationship were used as the dependent variable in a General Linear Mixed Model (GLMM) to test the effect of treatments during growth. We included both periods (first and second half of the experiment) in the analyses and tested for potential interaction between period and treatment. We followed the same procedure for head-bill (Gompertz function) and tarsus (logistic function). We performed General Linear Mixed Models (GLMM’s) to test treatment effect on thyroid hormones during growth, body condition index during adult periods as well as basal metabolic rate.

The random structure of the GLMM’s was determined through model selection procedures following Zuur, Ieno & Elphick, 2010^65^. Nestling ID and brood ID were considered as random factors to account for repeated measurements on the same individual during growth as well as the dependence of individuals belonging to the same clutch.

Kolmogorov–Smirnov tests were used for normality of the data. Explanatory variables were scaled using z-scoring (by subtracting the mean of the variable and dividing the result by the variable’s standard deviation). Scale Fat Mass Index at day 90 was log-transformed to fit normality. Starting from the most complete and biologically relevant model, we successively excluded non-significant variables starting from the most non-significant. Then, the selected model was compared to the null model using Akaike Information Criterion adjusted for small sample (AICc). Models with $\bigtriangleup _{AIC _{c}}$ $\leqslant$ 2 are considered similar. To assess treatment effect, we used ANOVA (type III). GLMM’s were performed using lme4 package version 1.1-18-1 with R (version 3.4.4 (2018-03-15))^64^.

## Supplementary information

Supplementary Information
