# Supplementary material for: Early life neonicotinoid exposure results in proximal benefits and ultimate carryover effects
Source: Sci Rep. 2021 Jul 27;11:15252. doi: 10.1038/s41598-021-93894-2 (PMC8316441; doi:10.1038/s41598-021-93894-2)
Supplement: Supplementary file 1 — Supplementary Information [file 41598_2021_93894_MOESM1_ESM.pdf]

Early-life neonicotinoid exposure results in short-term benefits and longer-term carry over effects

Thomas Zgirski[1,2*], Pierre Legagneux[2,3,4], Olivier Chastel[4], Lyette Regimbald[1], Louise Prouteau[4,5], Audrey Le Pogam[1,2,7,8], Hélène Budzinski[5], Oliver P. Love[6], and François Vézina[1,2,7,8]

[1]Université du Québec à Rimouski (UQAR), Canada
[2]Centre de la Science de la Biodiversité du Québec (QCBS), Canada
[3]Université Laval, Canada
[4]Centre d'Études Biologiques de Chizé (CEBC), France
[5]Université de Bordeaux UMR CNRS 5805 EPOC-OASU
[6]University of Windsor, Canada
[7]Centre d'Études Nordiques (CEN), Canada
[8]Groupe de recherche sur les environnements nordiques (BORÉAS), Canada
[*]thomas-zgirski@orange.fr

ABSTRACT

Neonicotinoids are insecticides widely used as seed treatments that appear to have multiple negative effects on Neonicotinoids are insecticides widely used as seed treatments that appear to have multiple negative effects on birds at a diversity of biological scales. Adult birds exposed to a low dose of imidacloprid, one of the most commonly used neonicotinoids presented reduced fat stores, delayed migration and potentially altered orientation. However, little is known on the effect of imidacloprid on birds growth rate despite studies that have documented disruptive effects of low imidacloprid doses on thyroid gland communication. We performed a 2x2 factorial design experiment in Zebra finches, in which nestling birds were exposed to a very low dose (0.205 mg.kg body mass^{-1}) of imidacloprid combined with food restriction during posthatch development. During the early developmental period, imidacloprid exposure resulted in an improvement of body condition index in treated nestlings relative to controls. Imidacloprid also led to compensatory growth in food restricted nestlings. This early life neonicotinoid exposure also carried over to adult age, with exposed birds showing higher lean mass and basal metabolic rate than controls at ages of 90-800 days. This study presents the first evidence that very low-dose neonicotinoid exposure during early life can permanently alter adult phenotype in birds.

Appendix A

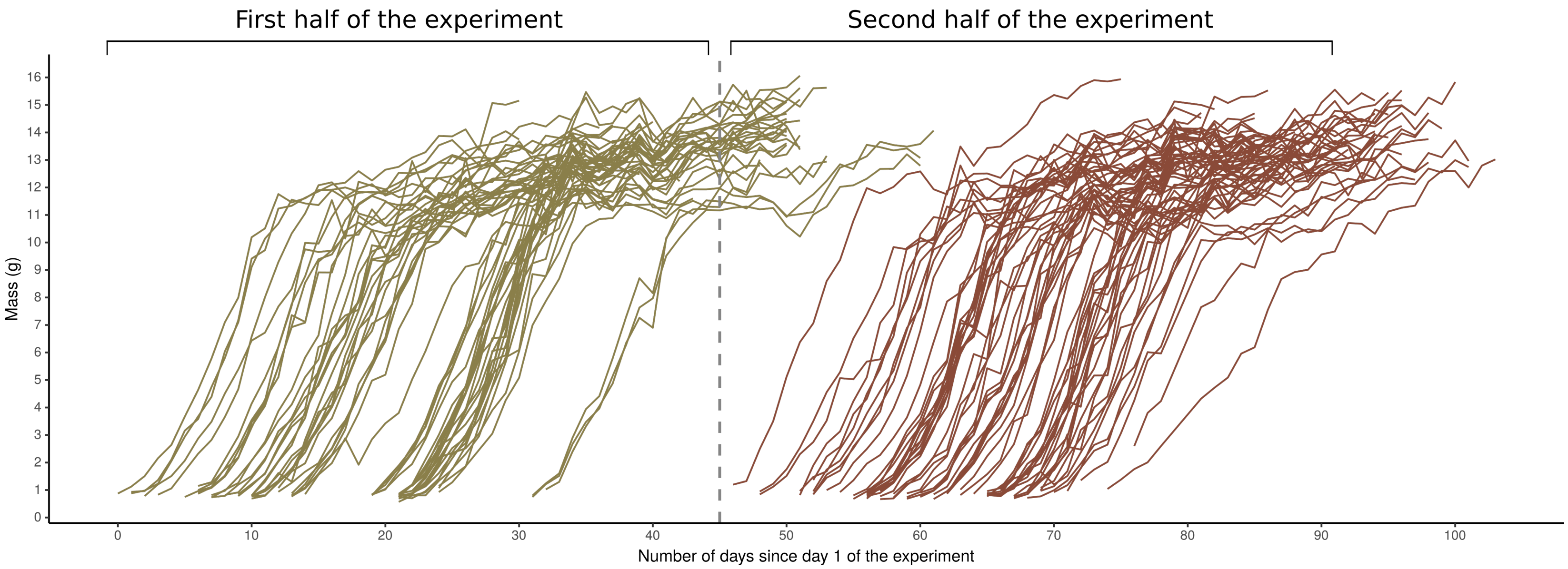

Complete presentation of all growth curves during the experiment according to time. Individuals in green (N = 60) and red (N= 69) were kept in the first and second half of the experiment respectively. Birds that hatched before 15/01/2017 were included in the first half of the experiment.

	Control	Imidacloprid
Ad libitum	30 (9)	38 (10)
Restriction	32 (9)	29 (8)

a) Sample size used in the experimental design for nestlings (and breeding pairs)

	First half		Second half	
	Control	Imidacloprid	Control	Imidacloprid
Ad libitum	18 (6)	13 (4)	12 (3)	25 (6)
Restriction	19 (6)	10 (3)	13 (3)	19 (5)

b) Sample size used in the experimental design split by period for nestlings (and breeding pairs)

Appendix B

Appendix B1

**a)** Model		N_p	$\triangle_{AIC_c}$	ω_i
Head-bill $_{residuals}$				
	1. Treatment*Period + Brood size + Sex	13	0.00	0.71
	2. Treatment*Period + Brood size + Sex + Pair order	14	1.84	0.28
	3. Null	4	8.57	0.01

**b)** Head-bill $_{residuals}$	β	*SE*	df	t-value	P-value
(Intercept)	-0.39	0.18	57.02	-2.11	≤ 0.05*
Ad libitum-Imidacloprid	0.12	0.17	34.59	0.71	n.s
Restricted-Control	-0.10	0.15	31.54	-0.65	n.s.
Restricted-Imidacloprid	-0.18	0.19	33.28	-0.98	n.s.
Periode	0.18	0.18	30.67	1.01	n.s.
Brood size	0.07	0.03	59.17	2.40	≤ 0.05*
Sex	0.16	0.07	120.85	2.29	≤ 0.05*
Ad libitum-Imidacloprid*Periode	-0.32	0.24	33.09	-1.31	n.s
Restricted-Control*Periode	-0.65	0.24	30.77	-2.65	≤ 0.05*
Restricted-Imidacloprid*Periode	-0.17	0.25	32.88	0.66	n.s.
Random effects					
Individual standard deviation	0.37				
Pair standard deviation	0.13				
Residual standard deviation	0.35				

a) Model selection of the effects of treatments (*Ad libitum*-Control, *Ad libitum*-Imidacloprid, Restricted-Control, Restricted-Imidacloprid), Sex, Brood size, Pair order on head-bill $_{residuals}$ from day 8 to day 30. **b)** Parameter estimates (slopes [β] with standard error [*SE*], degrees of freedom [df], t-value and P-value) for top ranked model. Reference level: *Ad libitum*-Control and Second half period. (N observations = 2937; from 129 individuals of 35 pairs used as random factors).

Appendix B2

**a)** Model		N_p	$\triangle_{AIC_c}$	ω_i
Tarsus $_{residuals}$				
	1. Treatment + Period	8	0.00	0.27
	2. Null	4	0.02	0.27
	3. Treatment	7	0.61	0.20
	4. Treatment + Period + Sex	9	1.07	0.16
	5. Treatment + Period + Brood size + Sex	10	2.75	0.07
	6. Treatment + Period + Brood size + Sex + Pair order	11	4.78	0.02
	7. Treatment*Period + Brood size + Sex + Pair order	14	6.60	0.01

**b)** Tarsus $_{residuals}$	β	*SE*	df	t-value	P-value
(Intercept)	0.26	0.11	27.54	2.31	≤ 0.05*
Ad libitum-Imidacloprid	-2.67	0.14	28.45	-1.87	n.s
Restricted-Control	-0.31	0.15	28.61	-2.09	≤ 0.05*
Restricted-Imidacloprid	-0.07	0.15	29.26	-0.43	n.s.
Periode	-0.18	0.10	29.60	-1.71	n.s.
Random effects					
Individual standard deviation	0.44				
Pair standard deviation	0.17				
Residual standard deviation	0.39				

a) Model selection of the effects of treatments (*Ad libitum*-Control, *Ad libitum*-Imidacloprid, Restricted-Control, Restricted-Imidacloprid), Sex, Brood size, Pair order on tarsus $_{residuals}$ from day 8 to day 30. **b)** Parameter estimates (slopes [β] with standard error [*SE*], degrees of freedom [df], t-value and P-value) for top ranked model. Reference level: *Ad libitum*-Control. (N observations = 1530; from 129 individuals of 35 pairs used as random factors).

Appendix C

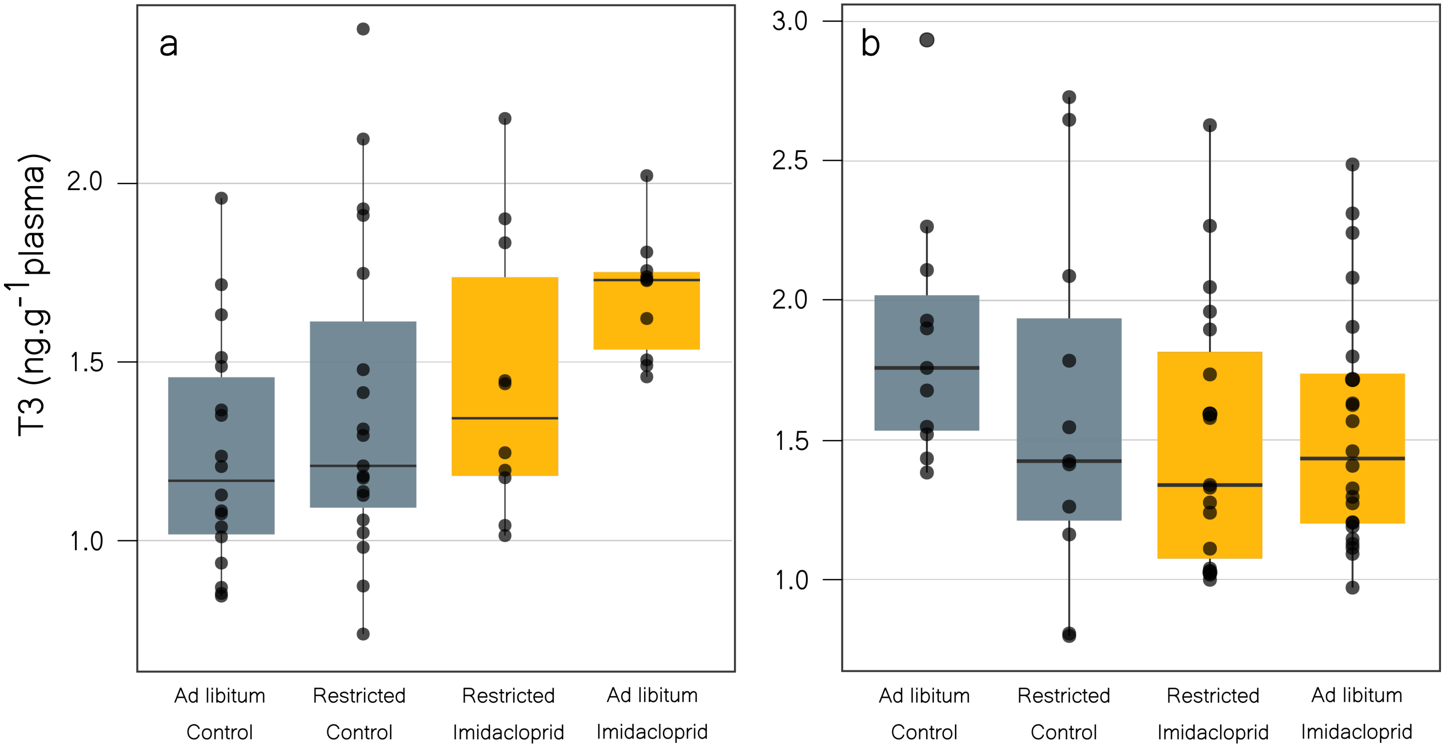

T3 concentration (ng.g^{-1} plasma) on day 15. a) First half of the experiment: *Ad libitum*-Control n=18, Restricted-Control n=19, Restricted-Imidacloprid n=10, *Ad libitum*-Imidacloprid n=10.) b) Second half of the experiment: *Ad libitum*-Control n=11, Restricted-Control n=11, Restricted-Imidacloprid n=19, *Ad libitum*-Imidacloprid n=24.

Appendix D

Appendix D1

**a)** Model	N_p	$\triangle_{AIC_c}$	ω_i
SMI_{90}			
1. Sex	4	0.00	0.51
2. Sex + Imidacloprid	5	0.30	0.44
3. Null	3	4.42	0.06

**b)** SMI_{90}	β	*SE*	df	t-value	P-value
(Intercept)	0.38	0.21	19.98	1.83	n.s.
Sex male	-0.58	0.22	49.44	-2.67	≤ 0.05*
Random effects					
Pair standard deviation	0.61				
Residual standard deviation	0.72				

a) Model selection of the effect of imidacloprid during the first half of the experiment (Imidacloprid; Control) and sex (Male; Female) on scaled mass index calculated and scaled at day 90 (SMI_{90}) (N_p = number of parameters, $\triangle_{AIC_c}$ = difference in AICc between the current and preferred model, ω_i = AICc weight in favor of the model) **b)** Parameter estimates (slopes [β] with standard error [*SE*], degrees of freedom [df], t-value and P-value) for top ranked model (N obs: 57; N pair: 16 (random factor))

Appendix D2

**a)** Model		N_p	$\triangle_{AIC_c}$	ω_i
SMI_{90}				
	1. Null	3	0.00	0.52
	2. Sex	4	0.59	0.38
	3. Food treatment + Sex	5	3.28	0.10

**b)** SMI_{90}	β	*SE*	df	t-value	P-value
(Intercept)	0.18	0.25	5.78	0.71	n.s.
Random effects					
Pair standard deviation	0.60				
Residual standard deviation	0.83				

a) Model selection of the effect of food during the first half of the experiment (*Ad libitum* ; restricted) and sex (Male; Female) on scaled mass index calculated and scaled at day 90 in control group (non imidacloprid exposed) (SMI_{90}) (N_p = number of parameters, $\triangle_{AIC_c}$ = difference in AICc between the current and preferred model, ω_i = AICc weight in favor of the model). **b)** Parameter estimates (slopes [β] with standard error [*SE*], degrees of freedom [df], t-value and P-value) for top ranked model (N obs: 36; N pair: 10 (random factor))

Appendix E

Appendix E1

**a**) Model		N_p	$\triangle_{AIC_c}$	ω_i
$SLMI_{90}$				
	1. Imidacloprid + Sex	5	0.00	0.99
	2. Null	3	9.02	0.01

$SLMI_{90}$	β	*SE*	df	t-value	P-value
(Intercept)	-0.10	0.19	19.67	-0.52	n.s.
Imidacloprid	0.97	0.27	13.51	3.52	≤ 0.001**
Sex male	-0.58	0.21	54.80	-2.72	≤ 0.001**
Random effects					
Pair standard deviation	0.32				
Residual standard deviation	0.75				

a) Model selection of the effect of imidacloprid during the first half of the experiment (Imidacloprid ; Restricted) and sex (Male; Female) on scaled lean mass index at day 90 ($SLMI_{90}$) (N_p = number of parameters, $\triangle_{AIC_c}$ = difference in AICc between the current and preferred model, ω_i = AICc weight in favor of the model). **b)** Parameter estimates (slopes [β] with standard error [*SE*], degrees of freedom [df], t-value and P-value) for top ranked model (N obs: 57; N pair: 16 (random factor))

Appendix E2

**a)** Model		N_p	$\triangle_{AIC_c}$	ω_i
$SLMI_{800}$				
	1. Imidacloprid + Sex	5	0.00	1.00
	2. Null	3	13.03	0.00

**b)** $SLMI_{800}$	β	*SE*	df	t-value	P-value
(Intercept)	0.01	0.26	16.94	0.04	n.s.
Imidacloprid	1.113	0.37	10.34	2.96	≤ 0.05*
Sex male	-0.85	0.21	26.20	-4.00	n.s.
Random effects					
Pair standard deviation	0.60				
Residual standard deviation	0.52				

a) Model selection of the effect of imidacloprid for birds from the first half of the experiment (Imidacloprid; Control) and sex (Male; Female) on scaled lean mass index calculated and scaled at day 800 ($SLMI_{800}$). (N_p = number of parameters, $\triangle_{AIC_c}$ = difference in AICc between the current and preferred model, ω_i = AICc weight in favor of the model). **b)** Parameter estimates (slopes [β] with standard error [*SE*], degrees of freedom [df], t-value and P-value) for top ranked model (N obs: 36 N pair: 15 (random factor))

Appendix F

Appendix F1

**a)** Model		N_p	$\triangle_{AIC_c}$	ω_i
$SLMI_{90}$				
	1. Sex	4	0.00	0.46
	2. Null	3	0.21	0.42
	3. Food treatment + Sex	5	2.70	0.12

**b)** SMI_{90}	β	*SE*	df	t-value	P-value
(Intercept)	0.25	0.24	13.56	1.03	n.s.
Sex male	-0.52	0.31	34.06	-1.70	n.s.
Random effects					
Pair standard deviation	0.45				
Residual standard deviation	0.84				

a) Model selection of the effect of food for birds from the first half of the experiment (*Ad libitum* ; restricted) and sex (Male; Female) on scaled lean mass index at day 90 in the control group (non Imidacloprid exposed) ($SLMI_{90}$). (N_p = number of parameters, $\triangle_{AIC_c}$ = difference in AICc between the current and preferred model, ω_i = AICc weight in favor of the model). **b)** Parameter estimates (slopes [β] with standard error [*SE*], degrees of freedom [df], t-value and P-value) for top ranked model (N obs: 36; N pair: 10 (random factor))

Appendix F2

**a)** Model	N_p	$\triangle_{AIC_c}$	ω_i
$SLMI_{800}$			
1. Sex	3	0.00	0.74
2. Null	3	3.31	0.14
3. Sex + Food treatment	5	3.57	0.12

**b)** $SLMI_{800}$	β	*SE*	df	t-value	P-value
(Intercept)	0.44	0.33	13.39	1.34	n.s.
Sex male	-0.92	0.33	16.53	-2.78	≤ 0.05*
Random effects					
Pair standard deviation	0.72				
Residual standard deviation	0.54				

a) Model selection of the effect of food for birds from the first half of the experiment (*Ad libitum* ; restricted) and sex (Male; Female) on scaled lean mass index at day 800 in control group (non Imidacloprid exposed) ($SLMI_{800}$). (N_p = number of parameters, $\triangle_{AIC_c}$ = difference in AICc between the current and preferred model, ω_i = AICc weight in favor of the model). **b)** Parameter estimates (slopes [β] with standard error [*SE*], degrees of freedom [df], t-value and P-value) for top ranked model (N obs: 20; N pair:9 (random factor))

Appendix G

Appendix G1

**a)** Model		N_p	$\triangle_{AIC_c}$	ω_i
$SFMI_{800}$				
	1. Imidacloprid	4	0.00	0.66
	2. Imidacloprid + Sex	5	2.67	0.17
	3. Null	3	2.84	0.16

**b)** $SFMI_{800}$	β	*SE*	df	t-value	P-value
(Intercept)	-0.38	0.26	10.31	-1.44	0.18
Imidacloprid	1.03	0.41	9.31	2.53	≤ 0.05*
Random effects					
Pair standard deviation	0.60				
Residual standard deviation	0.70				

a) Model selection of the effect of Imidacloprid for birds from the first half of the experiment (Imidacloprid; Control) and sex (Male; Female) on scaled-fat mass index calculated and scaled at day 800 ($SFMI_{800}$). (N_p = number of parameters, $\triangle_{AIC_c}$ = difference in AICc between the current and preferred model, ω_i = AICc weight in favor of the model). **b)** Parameter estimates (slopes [β] with standard error [*SE*], degrees of freedom [df], t-value and P-value) for top ranked model (N obs: 36 N pair: 15 (random factor))

Appendix G2

**a)** Model	N_p	$\triangle_{AIC_c}$	ω_i
$SFMI_{800}$			
1. Null	3	0.00	0.64
2. Sex	4	1.73	0.27
3. Sex + Imidacloprid	5	4.01	0.09

**b)** $SFMI_{800}$	β	SE	df	t-value	P-value
(Intercept)	-0.02	0.17	8.50	-0.11	n.s.
Random effects					
Pair standard deviation	0.42				
Residual standard deviation	0.90				

a) Model selection of the effect of Imidacloprid for birds from the second half of the experiment (Imidacloprid; Control) and sex (Male; Female) on scaled-fat mass index calculated and scaled at day 800 ($SFMI_{800}$). (N_p = number of parameters, $\triangle_{AIC_c}$ = difference in AICc between the current and preferred model, ω_i = AICc weight in favor of the model). **b)** Parameter estimates (slopes [β] with standard error [SE], degrees of freedom [df], t-value and P-value) for top ranked model (N obs: 43 N pair: 17 (random factor).

Appendix H

	First half		Second half	
	Control	Imidacloprid	Control	Imidacloprid
SFMI	16.21 (±1.85)	18.01 (±1.89)	17.13 (±1.73)	16.86 (±2.04)
SLMI	12.79 (±1.1)	13.85 (±1.02)	13.27 (±1.25)	13.60 (±1.19)

a) Mean (and standard deviation) of Scaled Fat Mass Index (SFMI) and Scaled Lean Mass Index (SLMI) according treatments and split by periods

Appendix I

Appendix I1

**a)** Model	N_p	$\triangle_{AIC_c}$	ω_i
BMR_{800}			
1. Imidacloprid + Lean mass	5	0.00	0.62
2. Null	4	2.00	0.23
3. Imidacloprid + Lean mass + Sex	6	2.86	0.15

**b)** BMR_{800}	β	*SE*	df	t-value	P-value
(Intercept)	-1.63	2.21	35.63	-0.74	n.s.
Imidacloprid	0.83	0.34	14.09	2.41	≤ 0.05*
Lean mass	0.09	0.17	35.55	0.56	n.s.
Random effects					
Pair standard deviation	0.17				
Residual standard deviation	0.85				

a) Model selection of the effect of Imidacloprid for birds from the first half of the experiment (Imidacloprid; Control) and sex (Male; Female) on scaled corrected basal metabolic rate at day 800 (BMR_{800}). (N_p = number of parameters, $\triangle_{AIC_c}$ = difference in AICc between the current and preferred model, ω_i = AICc weight in favor of the model). **b)** Parameter estimates (slopes [β] with standard error [*SE*], degrees of freedom [df], t-value and P-value) for top ranked model (N obs: 36; N pair: 15 (random factor))

Appendix I2

**a)** Model		N_p	$\triangle_{AIC_c}$	ω_i
BMR_{800}				
	1. Imidacloprid + Sex + Lean mass	6	0.00	0.56
	2. Lean mass + Sex	5	1.37	0.28
	3. Lean mass + Sex	4	2.69	0.15
	4. Null	3	9.33	0.01

**b)** BMR_{800}	β	*SE*	df	t-value	P-value
(Intercept)	-3.38	1.44	42.61	-2.38	≤ 0.05*
Imidacloprid	-0.59	0.28	8.16	-2.13	n.s.
Sex male	-0.54	0.24	34.19	-2.29	≤ 0.05*
Lean mass	0.30	0.10	41.85	2.91	≤ 0.001**
Random effects					
Pair standard deviation	0.51				
Residual standard deviation	0.69				

a) Model selection of the effect of imidacloprid during the second half of the experiment (Imidacloprid; Control) and sex (Male; Female) on scaled corrected basal metabolic rate at day 800 (BMR_{800}). (N_p = number of parameters, $\triangle_{AIC_c}$ = difference in AICc between the current and preferred model, ω_i = AICc weight in favor of the model). **b)** Parameter estimates (slopes [β] with standard error [*SE*], degrees of freedom [df], t-value and P-value) for top ranked (N obs: 43 N pair: 17 (random factor))
